# The political economy of sugar-sweetened beverage taxation in Latin America: lessons from Mexico, Chile and Colombia

**DOI:** 10.1186/s12992-020-00656-2

**Published:** 2021-01-05

**Authors:** Angela Carriedo, Adam D. Koon, Luis Manuel Encarnación, Kelley Lee, Richard Smith, Helen Walls

**Affiliations:** 1 World Public Health Nutrition Association, London, UK; 2 Department of International Health, Johns Hopkins Bloomberg School of Public Health, Baltimore, USA; 3 NCD Alliance, Mexico City, Mexico; 4 Faculty of Health Sciences, Simon Fraser University, Burnaby, Canada; 5 College of Medicine and Health, University of Exeter, Exeter, UK; 6 Faculty of Public Health & Policy, London School of Hygiene and Tropical Medicine, London, UK

**Keywords:** Political economy, Sugar-sweetened beverages, Taxation, Transnational corporations

## Abstract

**Background:**

In Latin America, total sales of sugar-sweetened beverages (SSBs) continue to rise at an alarming rate. Consumption of added sugar is a leading cause of diet-related non-communicable diseases (NCDs). Coalitions of stakeholders have formed in several countries in the region to address this public health challenge including participation of civil society organizations and transnational corporations. Little is currently known about these coalitions – what interests they represent, what goals they pursue and how they operate. Ensuring the primacy of public health goals is a particular governance challenge. This paper comparatively analyses governance challenges involved in the adoption of taxation of sugar-sweetened beverages in Mexico, Chile and Colombia. The three countries have similar political and economic systems, institutional arrangements and regulatory instruments but differing policy outcomes.

**Methods:**

We analysed the political economy of SSB taxation based on a qualitative synthesis of existing empirical evidence. We identify the key stakeholders involved in the policy process, identified their interests, and assess how they influenced adoption and implementation of the tax.

**Results:**

Coalitions for and against the SSB taxation formed the basis of policy debates in all three countries. Intergovernmental support was critical to framing the SSB tax aims, benefits and implementation; and for countries to adopt it. A major constraint to implementation was the strong influence of transnational corporations (TNCs) in the policy process. A lack of transparency during agenda setting was notably enhanced by the powerful presence of TNCs.

**Conclusion:**

NCDs prevention policies need to be supported across government, alongside grassroots organizations, policy champions and civil society groups to enhance their success. However, governance arrangements involving coalitions between public and private sector actors need to recognize power asymmetries among different actors and mitigate their potentially negative consequences. Such arrangements should include clear mechanisms to ensure transparency and accountability of all partners, and prevent undue influence by industry interests associated with unhealthy products.

## Background

There is now clear evidence that the excess consumption of added sugars, notably in sugar-sweetened beverages (SSBs), is associated with diet-related non-communicable diseases (NCDs) [1]. Taxing SSB has become an increasingly supported policy intervention for reducing the NCD burden, with 40 countries implementing such taxes by 2019 [2]. Five Latin American countries enacted legislation for a SSB tax between 2014 and 2018, including Mexico (January 2014), Chile (January 2015), Dominican Republic (September 2015), Ecuador (May 2016) and Peru (May 2018) [3]. Mexico and Chile were early adopters of SSB taxes and Colombia attempting implementation in 2015, even before it was defined as a “best buy” intervention by international organizations [4]. However, World Health Organization (WHO) recommendations regarding SSB taxation have elicited much debate [5–8]. Some evidence suggests the financial and health impacts of these policy instruments remain inconclusive [5–7, 9] while policymakers face varied challenges when designing health-related taxes [10–13]. Clouding public policy debate on this issue has been the substantial participation of vested commercial interests, notably large transnational corporations (TNCs) as SSB producers, whose profits are threatened by proposed fiscal measures [14]. Their political and economic power, across all levels of government [15], and the limited accountability and transparency mechanisms available to governments and civil society groups to monitor their undue influence, raises concerns about industry interference and conflicts of interest during the policy making process [6, 16–18]. In this context, it remains unclear how best countries can engage stakeholders in developing and implementing SSB taxes in the Latin American region and beyond. What are the optimal levels and structure of SSB taxation? How best might such taxes be framed to fit local political and economic contexts [17, 19]?

The aim of this paper is to contribute to this discussion through a critical review of the SSB taxation experience in Mexico, Chile and Colombia through a problem-driven political economy analysis. We describe how TNCs, through their economic and political power, have influenced the policy agenda on SSB taxation in these countries. The findings are used to identify broader lessons for protecting public health goals when developing and implementing fiscal policies that advance NCD prevention and control policies.

### The political economy of SSB production and consumption in Latin America

In 2012, the Latin American region became the world’s leading consumer of SSBs, contributing substantially to global growth in consumption over the previous decade [20]. A global analysis of the estimated daily caloric intake from SSBs per capita in 2015 found that four of the top ten countries were in Latin America: Chile (166 kcal/day/person), Mexico (158 kcal/day/person), Argentina (135 kcal/day/person), and Brazil (90 kcal/day/person) [21]. While economic hardship, due to high inflation and currency depreciation, have changed consumption levels in unpredictable ways in the region since 2015 [22, 23], the beverage industry continues to identify the Latin American region as a major SSB growth market [24].

TNCs involved in the production of SSB have been major investors in Latin America in recent decades, enabling them to capture a large market share in the region [23]. In 2018 the total revenues of Femsa Coca-Cola increased 6.8% over the previous year, reaching $23.9 billion (USD), while 11% of PepsiCo’s income ($7.04 billion USD) came from Latin America [25, 26]. From 2000 to 2013, sales of ultra-processed foods and SSBs increased from $38 billion (USD) to $81 billion (USD), larger than any other region (PAHO, 2015). In 2013 retail sales in Latin America of SSBs were 110.7 l/capita, with Mexico leading with 184.9 l/capita, followed by Chile 170.2 and Argentina with 156.1 l/capita, Uruguay with 123.7, Costa Rica 103.8 Guatemala 101.1 l/capita, while Colombia had 81.5 l/capita [27]. The Coca-Cola Company’s income before taxes in Latin America for 2016 amounted to approximately 1.97 billion (USD) with a retail value of $90 billion (USD), having a 48% value share in the beverage market [28, 29]. In addition, the market expansion and acquisition of smaller companies and bottlers in the region [22, 23] has expanded TNCs’ abilities to challenge regulatory measures that threaten their consolidated profits and power [23].

Strategic efforts by TNCs to influence policy decisions have been well documented for the food, beverage, alcohol and particularly tobacco industries [30–32]. A range of market (economic) and non-market (political) strategies have been employed, including constituency building, whereby relationships with key opinion leaders and policymakers in the community and health organizations are cultivated. TNCs, particularly SSB producers, have engaged in countries´ social and poverty alleviation programs, such as the provision of safe drinking water and nutrition education programs [22, 33]. Often these initiatives have taken the form of public-private partnerships (PPPs) or framed as corporate social responsibility (CSR) [34].

The participation of TNCs in PPPs and CSR initiatives, as multi-stakeholder initiatives, have previously raised questions about asymmetries of power in global health [35–37] and, more recently, in the political economy literature pertaining to Latin America [38, 39]. However, the existing literature on the participation of TNCs in the policy process related to SSB taxation in low and middle-income countries remains limited. Through analysis of three Latin American countries, this paper identifies lessons for pursuing similar disease prevention policies while mitigating, when required, the potential undue influence of TNCs on the policy process, and policy outcomes.

## Methods

We conducted a qualitative synthesis of document sources related to Mexico, Chile and Colombia between January 2011 and May 2018 (Updated in December 2019). A qualitative synthesis systematically searches for research on a topic, and draws the findings from individual studies together [40]. Case studies of Mexico, Chile and Colombia were selected because Chile and Mexico were the first two countries in Latin America (the region with the highest consumption of SSB globally) to have adopted SSB taxes, and Colombia is the only country in Latin America that has attempted and failed to introduce the tax (at the time of this review). The World Bank categorizes Mexico and Colombia as upper-middle-income economies [41] and Chile as a high-income economy [41]. They also share similar contextual factors such as type of government, geographic location and language, and they have similar obesity prevalence. In Mexico, 39% of adults are overweight and 33% are obese [42]. In Chile these ratios are 39 and 34% [43] and in Colombia 56% of adults are overweight and 19% are obese [44]. We selected three high-SSB consuming countries in the same region to identify common themes in the policy making process, focused on agenda-setting, to address an urgent health priority. Also, we sought to explain differences in the interaction between TNCs and health advocates during the policy process concerning SSB taxation, especially given that the TNCs operate across the three countries.

**Fig. 1:**
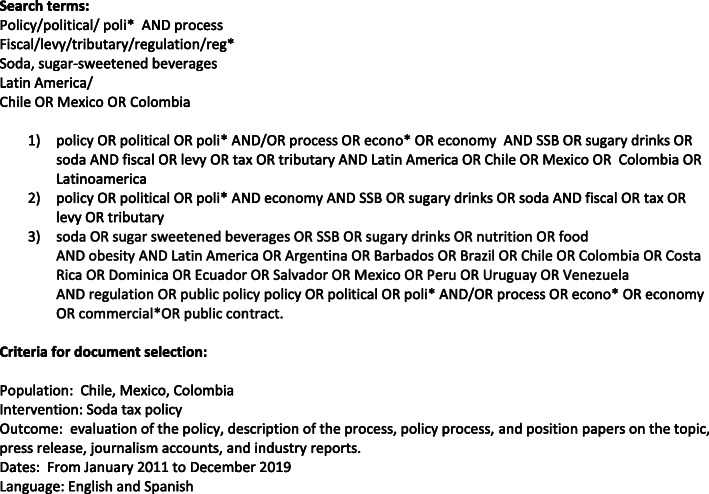
Search terms used for the documentary analysis and criteria to select documents

**Fig. 2:**
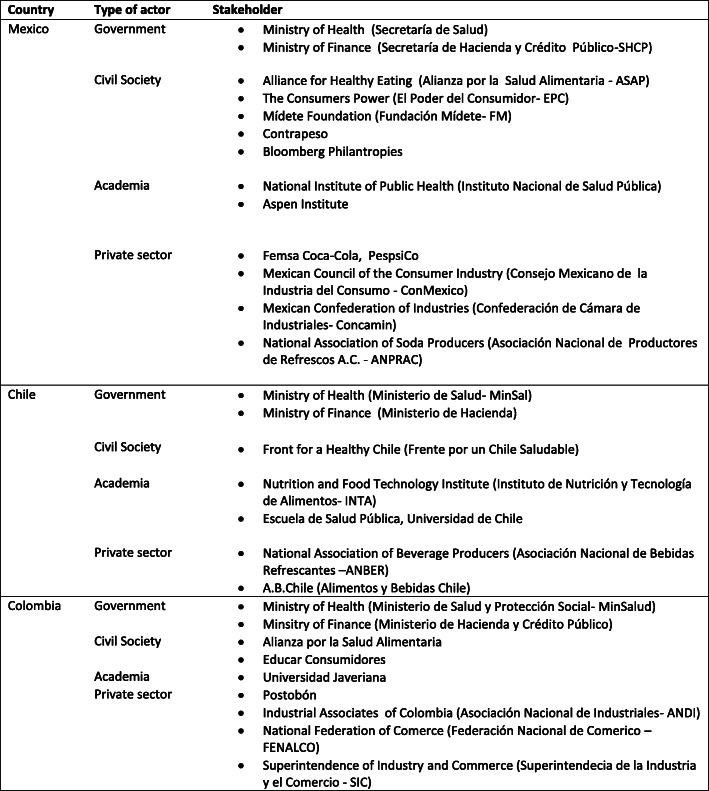
Stakeholders identified to be involved in SSB policy making by country

We began by conducting a systematic search of documents in English and Spanish. All articles were searched and screened by AC, with 10% screened by AK for validation purposes. No discrepancies needed to be addressed. Criteria for selecting documents followed a process similar to guidelines used in scoping reviews, defined as “an examination of a broader area to identify gaps in the research knowledge base, clarify key concepts, and report on the types of evidence that address an inform practice in the field” [45]. Documents were included if they were either in English or Spanish, and dated between January 2011 and December 2019 to cover the period when SSB tax policies were developed and implemented. Databases searched included Academic Complete, Scielo, Web of Science and Google Scholar using the same terms (Fig. 1). Backward reference searching was then applied to identify further documents. We also searched the websites of key stakeholders (identified after an initial screening of documents) to identify grey literature relevant to the analysis (listed in Fig. 2). Data included scientific publications (reviews, research articles, case studies, and commentaries), reports, newspaper articles, legal documentation and press releases by organizations and government officials generated before and during the soda tax design and/or implementation phases considered for inclusion literature related to the particular countries analyzed (January 2011 to December 2019). After removing duplicates, 35 peer-reviewed articles were included and 36 non-peer-reviewed documents (26 were reports either published by CSO or the SSB industry, 4 were legal documents, and 10 were either newspaper articles, press releases or website content).

**Fig. 3:**
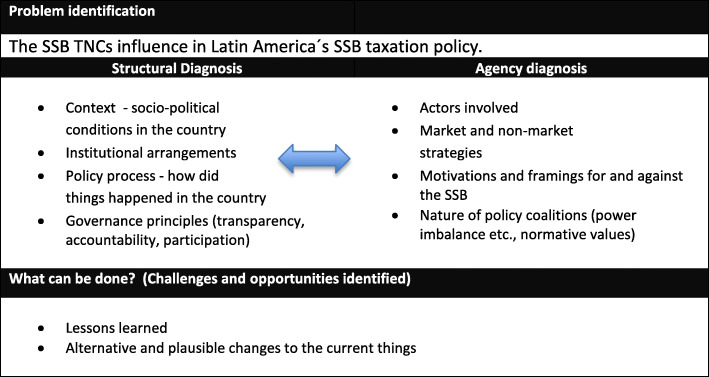
Analytical framework based on problem driven political economy framework Overseas Development Institute

The analysis and synthesis involved an iterative reflexive process by the authors, interpreting and developing meaning from the collected information, based on their expertise [46]. Findings were arranged as a narrative synthesis with the identified themes, guided by a framework put forth by the Overseas Development Institute to conduct applied political economy (PE) analysis [47]. We used this framework of problem-driven political economy (PE) because of its useful differentiation between structural features shaping an issue, including institutional factors, and features shaping an issue related to individual and organizational agency, including motivations, types of relationships and power dynamics among actors or stakeholders. Further, while providing conceptual rigor, this framework also has the flexibility and space to accommodate the concerns of our interdisciplinary research team, composed of researchers operating from different epistemologies. This problem-driven political economy analysis approach has three phases: a) problem identification, b) problem diagnosis; and c) considerations of the plausible change process (Fig. 3). We identified the problem as TNCs’ influence of SSB taxation policy in Latin America. The problem diagnosis includes issues of structure and agency described below in the results section [47]. Structural issues included the broader institutional arrangements shaping the relationships among key actors within the policy process, and the governance principles of transparency, accountability and participation. Agency issues included the political and business strategies of stakeholders seeking to frame policy debates for or against SSB taxes, (Fig. 3). We assessed how TNCs and local SSB producers influenced (or sought to influence) the design and implementation of SSB taxes with the main constraint of not following experts recommendation (20% tax minimum) to have a health impact. For this analysis, influence refers to the capacity of an individual or an organization to have an effect on the development or behaviour of someone or something. Finally, we addressed the last dimension of the PE framework by identifying challenges and opportunities, along with lessons learned, for introducing SSB taxation in other contexts.

## Results

### The role of structure and agency in SSB taxation policy debates.

#### Mexico

In 2012, results of the Mexican National Health and Nutrition Survey showed that 72.2% of the adult population was either obese or overweigh [48]. CSOs (civil society organizations) collectively started promoting SSB taxation in 2012 and proposing ways to increase it consecutive years. In 2013, following a year of strong civil society advocacy, President Enrique Peña Nieto launched the National Obesity and Diabetes Prevention Strategy. In 2013, a comprehensive Fiscal Reform proposed by the Ministry of Finance (MoF) also came into effect. Both policy instruments included a tax on SSBs and snacks with more than 250 kcal per 100 g (10 and 8% respectively). The final document was highly criticized by advocacy groups due to its argued loopholes; nevertheless, it was highly promoted by the government and accepted by the food and beverage industry (F&BI) after initial opposition [49].

The main proponent of the SSB tax was the Senate in December 2012, but it was rejected by the Congress (both Senate and Deputies chambers) in May 2013. That same year, the policy was overridden by the MoF as part of the President’s Fiscal Reform. A 1MXP per liter was accepted by Congress in October 2013. CSOs) advocating for the measure were *Alianza por la Salud Alimentaria*, *El Poder del Consumidor, Fundación Mídete* and the *ContraPESO Coalition,* all supported by Bloomberg Philanthropies. Academics from the National Institute of Public Health and Aspen Institute also engaged in policy debates and processes. TNCs and national SSB producers opposed the measure, and were represented by several business chambers including ConMexico, Concamin and ANPRAC (see Fig. 2).

An SSB tax was enacted in 2014 amid the convergence of several factors: a) evidence of poor results from self-regulatory measures for industry; b) high rates of obesity in the country; c) a new government administration seeking additional revenue sources; and e) an organized CSO advocacy campaign [18]. Notably, F&BI representatives also provided input on policy design, claiming their interests aligned with public health objectives. These factors positioned SSB taxation favourably on the policy agenda and facilitated its passage into law. Several key events took place after the enacting of the new tax in January 2014. The President agreed with ConMexico (industry consortium - Consejo Mexicano de la Industria de Productos de Consumo) that it would not increase the tax further after it came into effect; and a research institute established by the Coca-Cola Company was inaugurated by President Nieto, the health minister, and CEO of Coca-Cola Mexico. CSOs however advocated for a doubling of the tax [18].

After the tax was approved, the federal administration launched the Mexican Observatory on Non-communicable Diseases (OMENT – Observatorio Mexicano de Enfermedades No Transmisibles), as the Advisory Council delegated to monitor and evaluate the National Obesity and Diabetes Prevention Strategy. The Advisory Council included 20 representatives from the public sector, academia, professional organizations, civil society organizations, and industry-related representatives [50]. The two most influential of the latter were ConMexico, representing SSB producers, and the Aspen Institute Mexico, sponsored by and with strong ties to the SSB industry. Notably, none of the National Health Institutes were represented on the Council, nor were any of the consumer groups that had been instrumental in the promotion of the SSB tax. By the end of 2014, the OMENT had not produced reported on the impact of the SSB tax or even established indicators for assessing this impact. Meanwhile, an independent group led by the National Institute of Public Health, funded by Bloomberg Philanthropies, had reported a decrease in consumption [18, 51, 52].

According to the government, the Mexican SSB tax generated approximately US$1.2 billion in 2014 [53]. Although the regulation that introduced the tax designated funds (earmark) to increase access to clean water in schools, it remains unclear how the revenues were actually spent. In 2015, while advocating for an increase to the SSB tax, CSOs reported being harassed anonymously for their efforts [54].

#### Chile

In 2014, the Chilean MoF proposed the largest tax reform in three decades, with the aim of raising revenue for comprehensive educational reforms. Beverage taxes in Chile have existed since 1979, when the government introduced specific ad valorem taxes on alcoholic and non-alcoholic industrialized beverages, including SSBs. These beverages were initially subject to a 15% tax, reduced to 13% in 1985. The 2014 reform included a proposal to increase taxes on SSBs. The inclusion of a 20% tax on all SSB and an increased tax on all tobacco and alcohol products were advocated for by CSO groups through media campaigns, opinion pieces in newspapers, and public actions. Arguments were mainly health-related, focusing on the high consumption of SSB and 60% prevalence of obesity in the adult population [9]. Additionally, tax supporters highlighted the state’s responsibility to protect vulnerable populations through legislation [55].

The main proponent of the tax was the MoF through the fiscal reform process. In the beginning, the Ministry of Health (MoH) was indirectly involved through its work on similar regulations for healthy eating which included food labelling and marketing to children. CSO proponents of both initiatives were the Coalition for a Healthy Chile (*Frente por un Chile Saludable)*, Senator Guido Girardi, and academics from the *Institute of Nutrition and Food Technology* (Instituto Nacional de Nutricióny Tenconlogía Alimentaria INTA) and University of Chile. The private sector was mainly represented by A.B Chile, a national consortium of national and transnational food and beverage producers (See Fig. 2).

The F&BI contested the SSB tax proposal, based on similar arguments used by tobacco and alcohol industries, and in other SSB tax cases. They argued job losses, negative effects on the economy and trade, restriction of freedom of choice, regressivity (greater impact on the poorest groups) of the tax, the imposition of a ´nanny state´ and the ´arbitrary discrimination´ argument questioning the legality of the proposal [8]. Lobbying with congressional members intensified, and powerful coalitions were formed in opposition to the regulation. Few regulations were in place in the country to prohibit corporate lobbying and financing of political campaigns. Therefore some legislators and members of the MoF, heavily lobbied by industry interests, became opponents of the tax [56]. A coalition of stakeholders then aligned with the F&BI to reduce the SSB tax to 5%, far below the 20% recommended by CSOs to curb consumption (Table 1). This was further undermined by incorporating a tax exemption for some ‘low-sugar’ SSBs (less than 15 g per 240 ml) [57]. Thus, as happened in Mexico, the tax reform included amendments reducing the level of the tax and limiting its potential impact on consumption.

**Table 1:** Political and health context, regulations, content and reported impact; and institutional arrangements for the soda tax policies by country

Themes	Country		
	Mexico	Chile	Colombia
**Context**			
Political context	Entering president in 2013	Major tax reform in October 2014	Tax reform project (SSB and tobacco tax). Peace referendum with FARC
National obesity trends	37.8% overweight and 32.4% obesity among adults (70.2% combined prevalence) 36% overweight and obesity in children (5 to 10y) [1]	39.8% overweight and 34.4% obesity among adults (74.2% combined prevalence) [2]	37.7% overweight and 18.7% obesity among adults (55.8% combined prevalence) 24.4% overweight and obesity in children (5 to 10y) [3]
SSB retail sales in 2013 [4]	184.9 l/capita	170.2 l/capita	81.5 l/capita
**Regulatory instruments, content and reported impact**			
Regulation used to frame the policy	Fiscal reform (January 2014) Obesity policy	Fiscal reform (October 2014)	Obesity policy Fiscal reform
Type of tax and rate	Excise tax of 20%/ Excise tax of 1 MXP/l (≈ 10%)	Two-tired High sugar content (HSC): Ad Valormen 18% (> 6.25 g sugar/100 mL 20%) Low sugar content (LSC): Ad Valorem 10% (< 6.25 g sugar/100 mL)	Excise tax 20% None
Earmarked tax	No, but the Senate passed a resolution to allocate a proportion of the SSB to provide public schools with water fountains	NO	N/A
Impact reported after the implementation on SSB purchases.	Daily per capita purchases decreased by an average of 6% (− 12 mL/capita/day) of taxed SSB. Low socioeconomic status households had an average decline between 9 and 17% compared with pre-tax monthly trends of 2013 [5].	Monthly per capita purchases of taxed HSC SSB decreased by 3.4% by volume (95% CI −5.9−−0.9%) and 4.0% by calories (95% CI −6.3−−1.9%) The volume of household purchases of LSC SSB increased 10.7% (95% CI 7.5–13.9%)) [6].	NA
**Framing the SSBs tax**			
Framing the SSB tax rationale Framing the Earmarked tax revenue	Health related tax and revenue needs. To improve water provision in schools and parks.	Health related tax and revenue needs. To invest in a health reform.	Health related tax. To invest in programs to reduce obesity trends.
Normative values about the SSB Industry in the country	TNCs and SSB producers provide employment and economic growth to the country’s GDP. Partnerships with government.	Employment important for productivity, and economy of the country. Investment in technology.	Inter sectorial relationships with broadcast industry.
**Institutional arrangements and participation of non-state actors driving the SSB tax implementation**			
Governmental entity leading the initiative	Ministry of Finance (SHCP)	Ministry of Finance (MHCP)	Ministry of Health (MinSalud)
Non-state actors participating on the policy debates*	Ministry of Health (MoH), Academia (INSP Civil Society Organizations International organisations (Bloomberg philanthropies) National industry and beverage consortiums Transitional SSB producers	MoH, Academia Civil Society Organisations National consortium of non-alcoholic beverages Transitional SSB producers	Academia Civil Society Organisation Media Transitional SSB producers
*Outlined also in Fig. 2			

Simultaneously, an intense debate about implementing a regulatory framework began which included a restriction on marketing of unhealthy food to children, and front-of-pack warning labels informing consumers when a product is high in calories, sugars, fats and salt [58, 59]. After long discussions and pressure from F&BI lobbyists, the regulation finally came into effect in 2015 [60]. After the experience with the SSB tax, the *National Association of Beverage Producers* became *A.B. Chile*, and hired a former member of parliament and prominent politician to be its representative. Since the implementation of the law, TNCs have filed several lawsuits against the Chilean State challenging the legality of restricting their trademarks, cases which are still pending [60]. At the international level, TNCs supported by World Trade Organization (WTO) argued the new labeling violates several trade rules and is an obstacle to international trade, thus the labeling stayed as Chile argued for the basic right to protect human health [61] [62].

#### Colombia

Colombia’s political context is key to understanding the policy process involved in the promotion and, ultimately, rejection of the country’s proposed SSB tax. During 2015, the Minister of Health convened a group of experts to draft a series of proposals for a health tax to be included in a tax reform project and be presented to the Congress by 2016. The proposal included plans to increase the tobacco tax and to introduce a new SSB tax [63]. At the time, Colombia engaged in extensive public and policy debates around the government’s peace referendum with the Revolutionary Armed Forces (FARC), which was finally achieved in November 2016 [63]. The latter resulted in a convoluted political scenario and one that could be argued as influencing the rejection of the tax, as the policy agenda was highly focused on the peace referendum.

The initiative to introduce a SSB tax came from the MoH, and was supported by CSOs and coalitions such as *Educar Consumidores* and Colombian Alliance for Healthy Eating (*Alianza por la Salud Alimentaria Colombia),* and an alliance of several other CSOs who joined to support the new fiscal measure. Academics supporting the measure were based at *Universidad Javeriana*, while the main representatives of the TNCs and SSB producers included *Postobón*, ANDI, FENALCO and SIC (see Fig. 2).

A SSB tax was proposed in 2016, supported by the government and CSO groups, but was ultimately not approved. The CSO *Educar Consumidores* was the main advocate for a 20% tax on SBBs, just as was advocated n Mexico and Chile, but the tax was voted against after several months under Congress scrutiny. Similar to Mexico and Chile, intense industry lobbying of Congress was undertaken, and anonymous harassment of activists (proponents of the tax) was reported [64].

Public statements by the Chamber of the Beverage Industry representative denied the benefits of the SSB tax. Pro-industry members argued that the SSB tax would cause job losses among the poor and that “the impact is of great concern especially in those people living in rural areas where bottled drinks constitute the sole reliable source of water” [65]. Meanwhile the F&BI TNCs collaborated in PPPs and CSR initiatives such as the establishment of the International Energy Balance Network, led by the Coca-Cola Company, and recruitment of allies in the country [66]. The industry also provided drinking water in poor communities, in collaboration with other partners [67]. The drinking water availability as a subjacent causal path to an hydration issue related to high consumption of SSB goes beyond the soda tax policy. Many countries such as Mexico, Chile and Colombia have water spring concessions (use and exploitation) and the governance of water access has loopholes that favour TNCs [68, 69].

The SSB industry strongly lobbied against the tax. For example, in September 2016 the National Association of Businessman in Colombia (ANE) and *Postobón*, a local subsidiary of a SSB TNC, won an important lawsuit against the State [63]. This lawsuit demanded the Superintendent of Industry and Trade to withdraw an advocates’ media campaign on the negative effects of SSB tax, claiming that it presented false and misleading information. Additionally, during the spring and summer of 2016, the media debate intensified. The newspaper *Vice Colombia* published three opinion pieces supporting the measure, shortly before its editor was abruptly fired, increasing public demands for accountability. Polls conducted by CSOs showed that 70% of the population favoured the measure, and 42 of 268 members of Congress supported it [64]. However, after intense lobbying in late 2016, it was finally rejected by Congress. This case mirrored the other two cases but was unsuccessful, with no window for further discussion under the current political administration.

### The role of ideas, framing of SSB taxation and power dynamics.

#### Motivations and framing for and against SSB taxes

Important differences were found in understanding the ways in which values and evidence were used to motivate and frame policy design in each country. First, while the MoF drove the SSB taxation initiative in Mexico and Chile, in Colombia the main proponent was the MoH, with support from the CSO. While both the initiatives in Colombia and Mexico originated within MoH, and were framed as part of a comprehensive plan to tackle obesity, in Chile it was only included as part of a broader fiscal reform. These findings suggest that policy change was in part attributable to inter-ministerial synergy of the government in framing the policy debate. While the regulatory instruments were the same, framing the SSB tax as a health-related policy appears to have legitimized public discourse, although economic arguments were always needed. This is a core mandate of the MoH, not MoF, which potentially explains variations in frame sponsorship across the countries. The MoH in Chile and Mexico participated to a limited extent in drafting the SSB tax, in both cases the MoH supported the measure, although in Mexico the support came much after its approval in Congress, as Mercedes Juan, the Secretariat of Health had close links to the food industry [18].

Nevertheless, SSB taxation was framed beyond a public health rationale. In Mexico and Chile, SSB taxes were framed as a revenue generation mechanism [14, 18]. In Colombia, where the tax was largely framed as a health intervention, the need to raise additional revenue was not substantively communicated as it was in the other two countries, and largely failed to gain traction in a crowded political agenda.

Second, as shown in Table 1, the type and rate of the tax in each country varied, and all three failed to pass taxes of 20%, the minimum price increase considered by experts to have a substantial impact on obesity rates in a short span of time [70, 71]. While there is little evidence on how the final level and type of taxation were established (1MXP per litre in Mexico and a two-tier 5% in Chile), interference of the industry was reported in both cases. The rationale behind setting the level of the tax was not publicly available, and both taxes in Chile and Mexico were significantly less than the evidence-based simulations recommended [72, 73]. Still, there is evidence that this can change; under the new presidential administration (2018–2024), the Mexican SSB tax has been increased due to inflation from 1.17 MXP per litre to 1.26 MXP per litre, and might increase to 2.26 MXP per litre [74].

Third, of the three countries only Mexico explicitly outlined plans to evaluate the impact of the tax. This was accomplished by launching a multi-sectoral platform to report the impact of this and other policies included in the MoH obesity strategy (Table 1). Nevertheless, to-date, published impact evaluations of SSB taxes have only been conducted by externally-funded academics (Table 1). How government officials use this evidence is unclear. For instance, in Mexico the opaque governance of OMENT (which ceased operations in 2019), lacking transparency and accountability mechanisms, means that little is known about how these findings were received, managed or supported, or how F&BI representatives may have influenced the non-response.

In all three countries, legislation containing SSB taxes was vague on its evidentiary basis. These included: a) a lack of clarity around resource allocation using SSB revenues to accelerate health gains, b) missing justification for the chosen size of the SSB tax, c) an undefined plan for *multisectoral* policy implementation and/or evaluation; and d) in the specific case of Chile, rationale for increasing the existing staggered levy on SSBs, with a health-oriented purpose policy.

### The role of relationships and power in coalition building around SSB taxation

Our findings suggest that TNCs producing and selling SSBs have remained for the last 20 years a long in a powerful position in all the countries of study. For instance, the former Mexican President Vicente Fox (2002–2006) was previously the CEO of The Coca-Cola Company-Mexico, and it was during Fox’s leadership of Coca-Cola Mexico that it became Mexico’s top-selling soft drink, increasing Coca-Cola’s sales by almost 50% [75]. Mexican Coca-Cola-FEMSA (the largest Coca-Cola subsidiary in the world, which The Coca-Cola-Mexico is a shareholder with 28%) is one of the five largest contributors to the gross domestic product (GDP) with Bimbo, Gruma (both F&BI), Cemex, and Telmex. Coca-Cola-FEMSA and PepsiCo, either directly or through CONMEXICO or ANPRAC, have been involved with political institutions, such as the Centre for Beverage Innovation, opened in 2016 with the MoH and the Mexican President.

In Colombia, Postobón was one of the top 14 largest companies contributing to the economy; from 2016 to 2017, its income increased by 4.7%. The beverage company has many social programs, including a university and a large program to promote active lifestyles. It has been awarded by national and international institutions, such as the Swedish Business Network in Colombia and the Institute of Internal Auditors of Colombia and the Secretariat of Transparency of the Presidency, allowing the company to improve its reputation and open business opportunities in the region´ [76].

In Chile the main opponents to the SSB tax were members of the National Association of Beverage Producers (Asociación Nacional de Bebidas Refrescantes –ANBER), including Coca-Cola Andina (Embotelladora Andina y Embotelladoras Coca-Cola-Polar), Embonor, and CCU. In 2011, the association reported an increase in SSB consumption of 11.8%, described as related to “growing the economy by the increase in jobs opportunities” [77]. In 2014, just before the SSB tax was included in the fiscal reform, ANBER became A.B.Chile (Alimentos y Bebidas Chile), growing the consortium as Nestlé and Carozzi joined. To date, it is the country’s most powerful food and beverage group, representing more than 20 companies [78].

Coalitions formed against the SSB tax policy were mainly composed by TNCs and national SBB producers (which some were acquired by TNCs in the process), including business associations, confederations and trade organizations, and in some cases relations with academics or CSOs, as some of the boards of trustees’ or advisors were part of the F&BI [79] (Fig. 2). Part of their influence is likely attributable to their ability to leverage financial and strategic resources to position their views in the pubic domain. The representatives of such coalitions engaged in discourse around cooperation with public health aims, and built alliances with local and national government entities [80].

In contrast, powerful coalitions were also formed for the purpose of supporting the tax. They represented several CSOs and academics, mainly via the *Alianza por la Salud Alimentaria* (both in Mexico and Colombia) and by *Frente por un Chile Saludable* in Chile. In Colombia, CSOs were advised by some academics, but academics did not lead the call. In Mexico, by contrast, academics led research underpinning SSB taxation, supported the drafting of the bill, and assisted with advocacy efforts [18, 80]. In Chile, while well-known public health academics were supportive of the measure, they were mainly advocating for other policy measures, such as warning labels on snacks and beverages, and had a long-standing close relationship with some policy entrepreneurs in bringing the policy to the agenda-setting process. At the time of the policy debates, and agenda setting, some pro-tax groups were supported by international organizations, and prestigious US based academics, supporting the coalitions [81]. However, in Chile, the Nutrition and Technology Institute in Chile (INTA), a prestigious academic institution supporting the legislation, was undermined by undisclosed conflicts of interest that damaged its credibility [56].

In the cases of Mexico and Colombia, corporate interests influenced the media. In Colombia, the largest soda producer in the country owns the primary media outlet. Therefore advertising by CSOs supporting the tax was denied. Likewise, in Mexico, CSOs reported that the two main broadcasting corporations denied space for their campaign showing the amount of sugar in SSBs and other similar campaigns designed to support the measure. Regardless of a clear power imbalance surrounding the public policy debate between those who supported or opposed the tax, in Mexico, the pro-tax coalitions, led by civil society, maintained a powerful position in public opinion.

In Chile, debates centered around broader regulatory measures and the principles behind fiscal reform, with little focus on the specifics of a SSB tax. The primary frame sponsor for regulatory changes was the Senate head of the Health Commission, Guido Girardi, a media-savy spokesman of CSOs and academics [82]. Likewise, in Mexico, a Senator, Marcela Torres, advocated for the SSB tax, and built a strong coalition with *Alianza por la Salud Alimentaria*, academics and the country office of the Panamerican Health Organization (PAHO), by positioning the tax as a health measure on the policy agenda. In Colombia, CSOs gained important public support for the SSB tax through polls and social media, but policy entrepreneurs within the private sector were able to leverage Congressional contacts to successfully counter the measure.

## Discussion

This study provides important insights into how the problem of obesity has been defined, and the role of SSB taxation in addressing this problem in Latin America. We describe the importance of the political and economic context, the actors involved in the policy debates, the dynamic ways in which SSB taxes were framed, and the coalitions formed to mobilize vested interests. In doing so, our findings reveal the often opaque means by which TNCs can assert influence in the policy process. This raises important implications for the governance of TNCs when seeking to use fiscal measures to reduce consumption of health-harming products.

The ways in which SSB taxes were framed in each country was critical to their success. As a means of recruiting social values to make complex policy positions comprehensible, framing is an emerging subject of inquiry in the health policy process [83]. In all three countries, CSOs and academics emphasized social responsibility by raising concerns about the impact of SSB consumption on obesity and diabetes, as well as access to safe drinking water [84]. Civil society and grassroots groups wielded arguments about improving access to healthy food and safe drinking water to promote public revenue allocation towards health concerns. The main outcome of such arguments was widespread public support and further galvanizing coalitions of CSOs in all three cases. Nevertheless, in Mexico and Chile, the MoF was instrumental in framing SSB taxes as a fiscal measure, a finding consistent with similar research in Mexico and Chile [81]. In Mexico, such arguments gained purchase on the policy agenda as revenue funds were legally assigned to provide water fountains in schools [85, 86], but this was neither the case in Chile nor in Colombia.

Concurrently, in all cases TNCs used arguments about their legal rights and obligations, free choice, nanny state and freedom of intellectual property right, as they had done to oppose food-marketing policy in Chile [58], and reminiscent of the tobacco and alcohol industries’ strategies [87]. In Chile and Mexico, SSB companies argued against the tax on the basis of the right of free consumption, while in Colombia they applied litigation measures to CSOs advertising against consuming SBB outlining the health risk this implies. This is notable as it demonstrates the extent to which TNC influence is tied to framing in the media.

In these cases, TNCs were also able to negotiate directly with government regarding policy implementation, successfully subverting policy design so that levels of levels of SSB taxation were not aligned with the existing evidence-based recommendations, and some beverages were declared exempt from the tax. Others have observed these phenomena in Chile and Mexico [81] and the Philippines [88]. In Colombia, even when advocates managed to raise the topic in public debate, political tactics from the industry opposing SSB taxes were strong enough to prevent it from reaching the policy and legislative agenda. In all three cases, the power of TNCs influenced policy discussions and outcomes.

Despite recent progress, the regulatory environment continues to be a major obstacle for addressing unhealthy foods and SSB consumption in most countries. In response, TNCs are increasingly focusing on emerging economies such as in Latin America, East Asia and Africa, with hopes to influence regulatory actions [21]. Our research suggests that this is particularly true in Mexico, Chile, and Colombia. As with tobacco companies, SSB companies have faced unexpected regulatory changes in LMICs and have adapted to contain the damage [87]. Therefore, companies have embarked upon remediation actions by strengthening PPPs and corporate political activism through policy debate and building strong regional and international networks through consortiums or charitable organizations. These strategies allow local fiscal benefits to foreign investors and might have direct effects on consumption patterns and may reduce public health policy space, as has happened in Myanmar with Coca Cola investments [89]. Our findings concur with tactics used by the tobacco industry and by the food industry in other countries [89]. Furthermore, these cases reflect that including several government agencies and strengthening grassroots movements and CSOs, having key policy champions, and having a multi-sectorial approach to the measure, outlining it in several policy documents, are key elements for fiscal policies to successfully navigate the health policy process, and has been recognized as a key element for policy success in other case studies [12, 81, 88, 90].

**Fig. 4:**
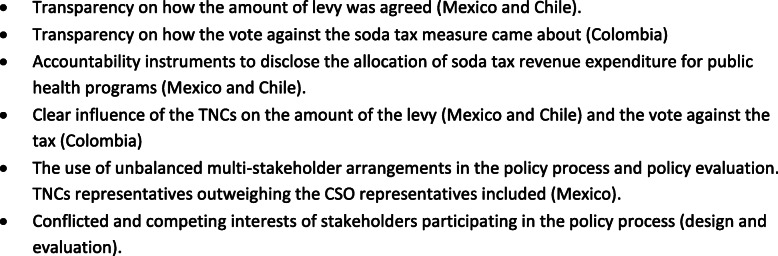
Governance gaps identified in the three cases

This study also found related concerns of transparency and accountability during policy design for SSB taxes in Mexico, Chile and Colombia. Modifications to the original proposals (20% tax) were not documented by official government sources. Secondary data documents suggest that TNCs influenced the final amount of the levy in both Mexico and Chile [8, 18]. Mechanisms to protect such influence lack in all cases, a clear governance loophole identified by others [37], which has not yet been addressed at the national or global level [15]. Additionally, how the revenues of the soda tax may support public health interventions is unclear. For instance, In Mexico some schools were provided drinking fountains by the PPPs established with Coca-Cola [91]. According to some evaluations in Chile, prices of untaxed beverages decreased, but taxed products did not sufficiently increase in price to reduce consumption. Nevertheless, the latest evaluation of the SSB tax found significant decreases in the volume of all soft drinks consumed and the monthly purchased volume of the higher-taxed sugary soft drinks by 21.6% [73]. These findings suggest principles of transparency and accountability during policy design and implementation were dismissed in general (Fig. 4).

Our findings challenge established discourse about private sector participation in policy design concerning harmful products. As a means of enhancing participation and generating consensus, the global governance discourse continues to advocate for NCD control through public-private (or multi-stakeholder) partnerships [4, 92]. Our research suggests, however, that TNCs distort public health agendas with an undue influence and by unfairly leveraging their resources to limit evidence-informed debate. This is particularly worrying for processes of institutionalization, whereby patterns of relationships can lead to further entrenched opposition to reasoned debate [93]. Moreover, public-private partnerships in health often involve powerful interests with conflicted aims that compete with public health prevention strategies [35].

The problem-driven approach to political economy analysis comprises three layers: identifying the problem, mapping the contextual, political and institutional arrangements around the SSB taxation and identifying the political economy drivers. This provides lessons about the obstacles, challenges and opportunities a SSB taxation initiative might face in similar contexts. It followed a qualitative synthesis based on a documentary analysis, which included a triangulation process between different sources to improve the reliability and validity of the information. The fact that several of our observations are shared by a similar analysis using primary data conducted by Fuster et al. [81] further underscores the utility of this approach. Nevertheless, some weaknesses were faced, such as the scarce public available information on the policy process for the countries included. For this reason, more in-depth research is needed that analyzes how stakeholders understand and shape the policy process for SSB taxation in Latin America.

This research points to pervasive gaps in global health governance. As TNCs, by definition, exist beyond state boundaries, governance of their activities and power must also occur at the global level. However, global health governance is highly challenged by contradictory and unclear guidelines by international organizations. Two principles of governance are at stake; responsiveness and ´participation and consensus´. Both principles lead to misinterpretation and open the door to powerful corporate interests to incisively participate in policy design. Additionally, some global health recommendations regarding policy actions to prevent NCDs, particularly regarding risk factors such as tobacco consumption and ultra-processed food availability, have evolved from broad recommendations to specific actions, and have recently focused more on engagement and governance rather than on policy implementation. This multi-country case study demonstrates the potential mechanisms for states to overcome TNC pressure.

## Conclusion

The aim of this paper is to better understand the governance challenges of ensuring the primacy of public health goals when designing and implementing SSB taxation. This is achieved by critically reviewing the experience of Mexico, Chile and Colombia using problem-driven political economy analysis. We identify lessons for developing and applying SSB taxation for NCD prevention goals. While it is important to consider a multi-sectorial approach when framing SSB taxation, and strong pro-tax coalitions were needed in all three countries to overcome entrenched opposition, these alliances must adhere to clear principles of transparency, accountability and participation. Importantly, our findings show how powerful industry-related actors seek to influence the policy process for SSB taxation, from agenda setting to implementation. TNCs producing and selling SSB have historically enjoyed positions of economic and political privilege in all three countries. Corporate coalitions have a powerful network of support in the region and resources to strategically position their views in the public domain to gain support. This includes industry representatives engaging in the discourse about PPPs and CSR with public health aims, based on alliances with local or national government entities. Efforts to advance SSB taxation thus need to carefully navigate vested interests shaping national and regional political economies. Countering the economic arguments of TNCs and other powerful industry actors can be achieved through adherence to good governance principles, including support by legal measures and broad alliances with CSOs, international actors and government entities.
